# Allelic drop-out may occur with a primer binding site polymorphism for the commonly used RFLP assay for the -1131T>C polymorphism of the *Apolipoprotein AV *gene

**DOI:** 10.1186/1476-511X-5-11

**Published:** 2006-05-02

**Authors:** Kirsten J Ward, Sian Ellard, Chittaranjan S Yajnik, Timothy M Frayling, Andrew T Hattersley, Prathyusha NS Venigalla, Giriraj R Chandak

**Affiliations:** 1 Institute of Biomedical and Clinical Science, Peninsula Medical School, Exeter, UK; 2 Diabetes Unit, King Edward Memorial Hospital and Research Centre, Pune, India; 3 Genome Research Group, Centre for Cellular and Molecular Biology, Hyderabad, India

## Abstract

*Apolipoprotein AV (ApoAV) *gene variant, -1131T>C, is associated with increased triglyceride concentrations in all ethnic groups studied. An *MseI *based RFLP analysis is the most commonly used method for genotyping this SNP. We genotyped a large cohort comprising 1185 Asian Indians and 173 UK Caucasians for -1131T>C using an ARMS-PCR based tetra-primer method. For quality control, we re-genotyped approximately 10% random samples from this cohort utilizing the *MseI *RFLP, which showed a 2.9% (3/102) genotyping error rate between the two methods. To investigate further, we sequenced the 900 bp region around the -1131T>C polymorphism in 25 Asian Indians and 15 UK Caucasians and found a number of polymorphisms including the -987C>T polymorphism. Further analysis of the -987C>T SNP showed a higher rare allele frequency of 0.23 in Asian Indians (n = 158) compared to 0.09 in the UK Caucasians (n = 157). This SNP is located 4 bp from the 3' end of the RFLP forward primer and is in weak linkage disequilibrium with -1131T>C variant (r^2 ^= 0.084 and D' = 1). Repeated RFLP analysis of seven subjects heterozygous for -987C>T (seven times), showed discordant results with the sequence at -1131T>C SNP nearly one third (15/49) of the time. We conclude that presence of -987C>T polymorphism in the forward primer of the *MseI *RFLP assay may lead to allelic drop-out and generate unforeseen errors in genotyping the -1131T>C polymorphism. Our results also emphasise the need for careful quality control in all molecular genetic studies, particularly while transferring genotyping methods between various ethnic groups.

## Introduction

Apolipoprotein AV is an important regulator of triglycerides concentrations. The *ApoAV *gene was identified adjacent to the Apolipoprotein cluster (consisting of *ApoAI*, *ApoAIV *and *ApoCIII *genes) [1,2]. The gene encodes a protein that is thought to be fundamental in the transport of triglyceride rich lipoproteins from the liver [3]. Its hepatic expression has been shown to influence post-prandial triglyceride concentrations [3] and has the potential to act as an intracellular break in the very low density lipoprotein (VLDL) assembly [4]. In addition, ApoAV also enhances VLDL metabolism through physical interaction with lipoprotein lipase [5].

Polymorphisms in *APOAV *are known to influence the circulating triglyceride concentrations. These variants are split into 3 common haplotypes, of which 2 are associated with increased triglyceride concentrations irrespective of age, gender, ethnicity, pregnancy, lipid profile, and dietary fat intake and are tagged by two SNPs, -1131T>C and S19W [1,6-9]. In keeping with this, the prevalence of the rare allele for these polymorphisms is higher among subjects with diseases associated with a raised triglyceride concentration such as Familial Hypercholesterolemia, Familial Combined Hyperlipidaemia and Hypertriglyceridaemia [10-15]. The rare allele at -1131T>C polymorphism is also associated with increased risk of coronary heart disease [16-18].

The rare allele frequency varies between different ethnic groups and populations of Chinese and Japanese descent have a higher prevalence of the -1131C allele and a lower prevalence of the W19 allele when compared to Caucasian populations [1,7,8,19,20].

This paper concentrates on a potential pitfall in the most widely used method (>65% publications) for genotyping the -1131T>C polymorphism using the abolition of a restriction site for *MseI *enzyme [1]. We detected an unacceptable error rate using our standard quality control checks (a random 10% of samples re-genotyped using a second method). Investigation of the discordant results between the RFLP and the tetra-primer methods for genotyping -1131T>C polymorphism led to identification of a polymorphism within the primer binding site of the RFLP forward primer resulting in allelic drop-out. To alleviate these problems, we propose alternative methods for genotyping -1131T>C polymorphism in the *APOAV *gene.

## Results

### Discordant results in samples typed by two methods

One thousand, one hundred and eighty five Asian Indian and 173 UK Caucasian subjects were initially genotyped for the -1131T>C polymorphism using the tetra-primer ARMS-PCR method. In keeping with our standard laboratory protocol, 102 of these subjects were re-genotyped using the *MseI *RFLP as a quality control check. In 2.9% (3/102) of samples the results for the two methods were discordant. This high rate of discordant samples could not be explained by sample mix-up or by failed or partial digestion. Sequencing suggested that the erroneous results were due to the RFLP method.

### Polymorphisms present in the primer binding sites

**Figure 1:**
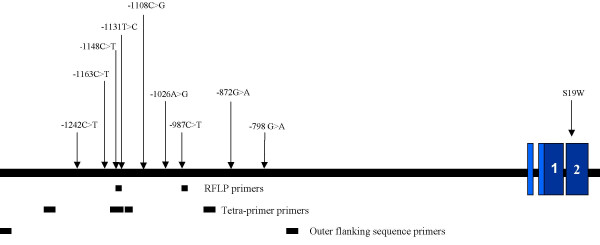
Schematic of the *ApoAV *gene, its variants and primer positions.

**Figure 2:**
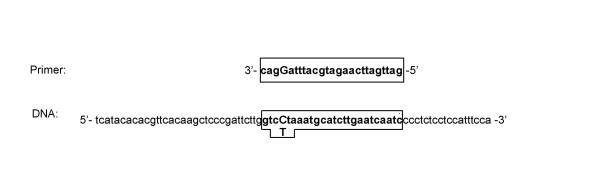
This shows the -987C>T polymorphism which potentially alters primer binding for the RFLP method of genotyping (primer sequence is placed within the boxed area, with the site of the polymorphism in capital letter).

As the discordant results could most plausibly be explained by allelic drop-out, we sequenced the 900 bp region covering the primer sequences for both the methods in 40 subjects (25 Asian Indians and 15 Caucasians). Sequence analysis identified several polymorphisms (Fig. 1) of which three were located in the primer binding sites and may potentially alter primer binding. The -987C>T (rs17120035) polymorphism was situated 4 bp from the 3' end of the forward primer for RFLP analysis (Fig. 2). Two other polymorphisms were identified in the tetra-primer inner primer regions; -1108C>G (rs1729411) was 5 bp from the 5' region of the forward primer and a novel SNP -1148G>A that was located 9 bp from the 5' end of the reverse primer (Fig. 1).

Since no polymorphisms were identified in the region of the outer primers used for the tetra-primer assay, we used them for sequencing to assess the prevalence of the identified polymorphisms in 158 unrelated Asian Indian adults and 157 unrelated UK Caucasian adults (table 1). The -987C>T polymorphism located within the RFLP forward primer was fairly common with a rare allele frequency of 0.23 in Asian Indian subjects and 0.09 within UK Caucasian subjects. While the -1108C>T SNP was not observed in the Asian Indian subjects and only in the UK Caucasian subjects (rare allele frequency = 0.10), the -1148C>T polymorphism situated within the tetra-primer inner reverse primer was observed in the Asian Indian samples only (rare allele frequency = 0.01). The polymorphisms studied were in Hardy Weinberg Equilibrium (HWE) and linkage disequilibrium was observed between -987C>T and -1131T>C both in Asian Indians and UK Caucasians (D' = 1.00, r^2 ^= 0.084 and D' = 0.998, r^2 ^= 0.001, respectively).

**Table 1:** Rare allele frequencies for the polymorphisms in the *ApoAV *gene found through initial sequencing

Polymorphism	Refseq number	Asian Indian	UK Caucasian
-798G>A	rs10750097	0.27	0.27
-872A>G	rs7103224	0.20	0.07
-987C>T	rs17120035	0.23	0.09
-1026G>A	Not on databases	0.35	0.00
-1108C>G	rs4938312	0.00	0.10
-1131T>C	rs662799	0.22	0.05
-1148C>T	Not on databases	0.01	0.00
-1163C>T	rs1729411	0.05	0.02
-1242C>T	Not on databases	0.004	0.01

### Heterozygosity in the primer polymorphism -987C>T is associated with errors in genotyping -1131T>C using the RFLP assay

We performed the *MseI *RFLP assay for -1131T>C on two separate occasions in 82 Asian Indians of known genotype (by sequencing the SNP). This showed at least one discordant result between the sequencing and the RFLP in 13 subjects (14%), with three being discordant on both occasions. Eight of the 13 (62%) discordant samples were heterozygous for both the -1131T>C and the -987C>T polymorphisms compared to 1 of the 69 (1.4%) who did not show discordant results (p < 0.001). As heterozygosity at the -987C>T polymorphism was associated with discordant results, this suggests that this polymorphisms altered primer binding resulting in allelic drop-out on RFLP assay for -1131T>C. To substantiate the role of -987C>T SNP further, we identified 7 subjects heterozygous for both -1131T>C and -987C>T and re-genotyped them using the RFLP assay 7 times. Results for the RFLP differed with sequencing (gold standard) results for 15 out of a total of 49 genotypes (31%) with each subject being discordant on at least one occasion. No individual showed a genotype that was different from that determined by sequencing on all 7 occasions (range 1/7 – 4/7). These results suggest that -987C>T SNP causes allelic drop-out on some but not all occasions.

## Discussion

Our study shows that presence of a polymorphism-987C>T in the *ApoAV *gene can produce allelic drop-out resulting in an incorrect genotype at SNP -1131T>C when genotyping by the widely used *MseI *RFLP assay. The error occurs in the background of heterozygosity both at -987C>T and -1131T>C and only on some occasions on the same sample. This potential source of error was probably detected in an Asian Indian cohort because both -987C>T and -1131T>C are over 3 times more prevalent than UK Caucasians. There is evidence in the literature that common polymorphisms within primer binding sites [21-23] can result in allelic drop-out which may be due to preferential amplification of one allele or through other mechanisms [24]. The degree of allelic drop-out may also result in a relative lack of heterozygous subjects within a population and deviation from Hardy Weinberg equilibrium [23]. Many such polymorphisms are known to be race-specific [25]. As the majority of data on -1131T>C exists on European Caucasian subjects with very limited data available on other ethnic groups such as Asian Indians and Sub-Saharan Africans, our observations prompt for more caution while initiating similar analysis in new populations.

The -987C>T polymorphism is likely to contribute to genotyping error especially in Asian Indian samples if the previously described primers are used. Therefore it is wise to use another assay to define the genotype at -1131T>C such as the ARMS-PCR based tetra-primer assay we used [9]. This is based on a method first described by Ye et al, in 2001 [26] with primers designed using the Tetra-primer ARMS-PCR website[27]. Although we did find SNPs that are present in primers used in this method, these are considerably less common, are not close to the 3' end of the primer and no errors were seen compared to sequencing. Therefore the tetra-primer method is our preferred alternative.

We have developed a new restriction assay for the -1131T>C polymorphism that avoids all known polymorphisms in primer binding sites. This uses the tetra-primer outer forward primer (5'-CAAGGTGACAGACAACTGGTGCAATGAT-3') and the RFLP reverse primer (5'- CCCCAGGAACTGGAGCGAAATT-3') to amplify a fragment of 239 bp. In the presence of the rare C allele, *MseI *digestion will result in fragments of 217 bp and 22 bp. The PCR was performed in 10 μl reactions with standard reagents using 2.5 mM MgCl_2 _and 0.5U of Amplitaq Gold (Applied Biosystems, UK) using cycling conditions as initial denaturation at 94°C for 12 minutes followed by 40 cycles of denaturation at 94°C for 45 seconds, annealing at 63°C for 45 seconds and extension at 72°C for 45 seconds, with a final extension of 72°C for 10 minutes. The *MseI *digestion was performed as previously described by Pennacchio et al, 2001 [1]. This new PCR RFLP assay worked in 96 samples with no discrepancies and no errors when compared to the original genotyping results for the tetra-primer assay.

## Conclusion

In conclusion, the -987C>T polymorphism is present in UK Caucasians and Asian Indians and can result in allelic drop-out within the widely used *MseI *RFLP assay for genotyping the triglyceride associated polymorphism -1131T>C. This is especially important in the Asian Indian population as both -987C>T and -1131T>C polymorphisms are more prevalent. Our result stresses the importance of checking and repeatedly checking SNP databases for polymorphisms in the primer binding sites (the -987C>T polymorphism was not reported when we started out studies on the -1131T>C polymorphism). It also emphasises the need to use a second genotyping method as a quality control. This type of check is particularly important when studying new populations as allele frequencies of the same polymorphism can vary greatly between populations meaning a primer binding site polymorphism may be of much greater significance in some populations than others.

## Materials and methods

Genomic DNA samples from 1185 Asian Indian subjects from 395 Asian Indian family trios (mother, father and child) from the Pune Children Study [28,29] and 173 unrelated UK Caucasians from the EFS and Plymouth EarlyBird study [9,30] were initially genotyped for -1131T>C variant using an ARMS based tetra-primer method. The design of primers would amplify a control product of 404 bp while the PCR products of 250 bp and 242 bp would identify T and C alleles respectively.

The primer sequences used are as follows:

Tetra-primer Outer Forward: 5'-CAAGGTGACAGACAACTGGTGCAATGAT-3'

Tetra-primer Outer Reverse: 5'-AGCCCCTGAAAGCTTCACTACAGGTTCC-3'

Tetra-primer Inner Forward: 5'-TTCAGCTTTTCCTCATGGGGCAAATATC-3'

Tetra-primer Inner Reverse: 5'-GAGCCCCAGGAACTGGAGCGAAATTA-3'

The PCR was performed using cycling conditions of initial denaturation at 95°C for 2 min, followed by 35 cycles of denaturation at 95°C for 1 min, annealing at 59°C for 1 min and extension at 72°C for 1 min, with a final 2 min extension at 72°C. PCR products were run on a 3% agarose gel at 200 V for 90 minutes and scored by two independent workers (KW and GRC). Subsequently, ~10% of these samples (n = 102) were analysed by PCR-RFLP using 2U of *MseI *enzyme [1] for the quality control check.

In an attempt to investigate the discordant results between the RFLP based and tetra-primer based genotyping, primers were designed to amplify a 900 bp region encompassing both tetra-primers and RFLP primers (Fig. 1). Finally, tetra-primer outer primer pair was utilised as the gold standard for confirmation of individual sequence since this region did not show any polymorphism on sequence analysis of the 900 bp region. All the PCR products were purified using post-PCR purification plates from Millipore and sequenced individually on both the strands using Big Dye terminator cycle sequencing ready kit (Version 1.1, Applied Biosystems, Warrington, UK) on ABI 3100 Genetic Analyzer (Applied Biosystems).

## Abbreviations

ApoAV – Apolipoprotein AV

ApoAI – Apolipoprotein AI

ApoAIV – Apolipoprotein AIV

ApoCIII – Apolipoprotein CIII

VLDL – Very Low Density Lipoprotein

SNPs – Single Nucleotide Polymorphisms

RFLP – Restriction Fragment Length Polymorphism

PCS – Pune Children Study

EFS – Exeter Family Study of Childhood Health

PEB – Plymouth EarlyBird study

HWE – Hardy Weinberg Equilibrium

LD – Linkage Disequilibrium

PCR – Polymerase Chain Reaction

## Competing interests

The author(s) declare that they do not have any competing interests.

## Authors' contributions

KJW carried out the molecular genetic studies, performed statistical analysis and drafted the manuscript. SE advised on methodologies. CSY designed the studies of the phenotype of patients and supervised the isolation of DNA. TMF and ATH helped advise on design of molecular studies. GRC designed and carried out molecular genetic studies. PNSV performed the molecular genetic studies especially the sequencing. All authors read and approved the final manuscript.
